# CGP42112: the full AT_2_ receptor agonist and its role in the renin–angiotensin–aldosterone system: no longer misunderstood

**DOI:** 10.1042/CS20220261

**Published:** 2022-11-03

**Authors:** Yazmin M. Restrepo, Natalia M. Noto, Robert C. Speth

**Affiliations:** 1 Department of Pharmaceutical Sciences, College of Pharmacy, Nova Southeastern University, Fort Lauderdale, FL 33328, U.S.A.; 2 Department of Physiology and Pharmacology, School of Medicine, Georgetown University, Washington, DC 20007, U.S.A.

**Keywords:** Antagonist, AT2R, CGP42112, Full Agonist, Partial Agonist

## Abstract

For years, the AT_2_R-selective ligand CGP42112 has been erroneously characterized as a partial agonist, partly due to its ability to also interact with the AT_1_R at high concentrations. As late as 2009, it was still being characterized as an antagonist as well. In this perspective/opinion piece, we try to resolve the ambiguity that surrounds the efficacy of this compound by extensively reviewing the literature, tracing its beginnings to 1989, showing that CGP42112 has never been convincingly shown to be a partial agonist or an antagonist at the AT_2_R. While CGP42112 is now routinely characterized as an AT_2_R agonist, regrettably, there is a paucity of studies that can validate its efficacy as a full agonist at the AT_2_R, leaving the door open for continuing speculation regarding the extent of its efficacy. Hopefully, the information presented in this perspective/opinion piece will firmly establish CGP42112 as a full agonist at the AT_2_R such that it can once again be used as a tool to study the AT_2_R.

CGP42112 is a peptidomimetic compound [1] that functions as an angiotensin II type 2 receptor (AT_2_R)-selective ligand. It has a long and complex history of differential characterization, surrounded by much ambiguity and uncertainty. A PubMed search of the word ‘CGP42112’ on July 12, 2022 found a total of 329 publications. Another 20 publications were found as derivatives of the original search. Among these manuscripts, there are many that contribute to the ambiguity that surrounds CGP42112’s efficacy. They can be divided into two groups: one of research papers, which present data to demonstrate or infer antagonistic, partial agonistic or full agonistic classifications at the AT_2_R, and another group of review papers or research studies that did not directly investigate CGP42112 but contribute to the ambiguity indirectly (by citing other authors) or by merely presenting the concept of partial agonism or antagonism without substantiation. We selected 196 of these papers as representative of the categorization of the efficacy of CGP42112 at the AT_2_R. We did not cite every paper that had CGP42112 in its text as many papers are reviews of the entire renin–angiotensin–aldosterone system (RAAS) and CGP42112 actions at the AT_2_R were not a major focus of such papers.

First discovered by Whitebread et al. at Ciba-Geigy in 1989, CGP42112 was originally developed to be an angiotensin (Ang) II receptor antagonist (Supplementary Table S1). However, the present study did not assess the efficacy of CGP42112, as it simply showed it to be a selective ligand of the Ang II receptor subtype ‘A’ (now known as the AT_2_R) [2]. However, 11 of the 89 citations, which we include in Supplementary Table S1, cite it as the basis for characterizing CGP42112 as an antagonist. A subsequent report [3] which characterized CGP42112 as an antagonist of the ‘angiotensin IIB receptor subtype’ (now known as the angiotensin II type 1 receptor, AT_1_R) may have also added to the perception that CGP42112 was an AT_2_R antagonist. It is noteworthy that the 1991 ‘Report of the Nomenclature Committee of the Council for High Blood Pressure Research: Nomenclature for Angiotensin Receptors’ reported that CGP42112A was an AT_2_R antagonist, but with a caveat that until an established functional response mediated by the AT_2_R was established, that it was not possible to make a definitive determination of its efficacy. Indeed, this caveat was prescient when the functional response of the AT_2_R as a physiological antagonist of the AT_1_R was recognized, such that the antagonism of AT_1_R mediated responses by CGP42112 could be recognized as an AT_2_R agonist response. Later, it was shown to be an AT_2_R agonist mediating an inhibition of T-type calcium channels that mimicked that of Ang II in cells expressing only AT_2_R, strongly suggesting that it was a full agonist but not providing definitive data [4]. In 1993, some of these same investigators [5] did characterize CGP42112 as a full AT_2_R agonist showing it to be equiefficacious with Ang II at decreasing cyclic guanosine monophosphate (cGMP) levels by inhibiting particulate guanylate cyclase, and years later Widdop et al. confirmed the full agonism of CGP42112 at the AT_2_R [6]. This should have been the definitive characterization of the efficacy of CGP42112 as a full agonist, despite its initial characterization as an antagonist. However, as noted below, sometime later the characterization of CGP42112 began to be misconstrued (Supplementary Tables S1 and 2). Different papers started referring to CGP42112 as an antagonist of the AT_2_R (Supplementary Table S1), or as a partial agonist or a partial antagonist of the AT_2_R (Supplementary Table S2), leading to the uncertainty that still surrounds the characterization of this compound.

## AT_2_R signaling and functionality

The AT_2_R is well characterized structurally as a G-protein-coupled receptor (GPCR) that is abundantly present during the fetal development, but has reduced expression during adult life, when the AT_1_R becomes predominant [7–9]. While the AT_1_R mediates most of the physiological and pathophysiological effects of Ang II in tissues such as vascular smooth muscle, endothelium, heart and kidney [10], the AT_2_R is thought to oppose its effects by inhibiting cell proliferation and differentiation, promoting vasodilation, while reducing inflammation and oxidative stress [11,12]. Under controlled conditions, when AT_1_R function is blocked, stimulation of the AT_2_R by Ang II lowers blood pressure [13,14], and causes vasodilation [15], although these effects are not observed in all studies [16,17]. Thus, the physiological significance of the AT_2_R is still not fully understood, contributing to its ambiguity and that of CGP42112 as an AT_2_R agonist.

**Figure 1:**
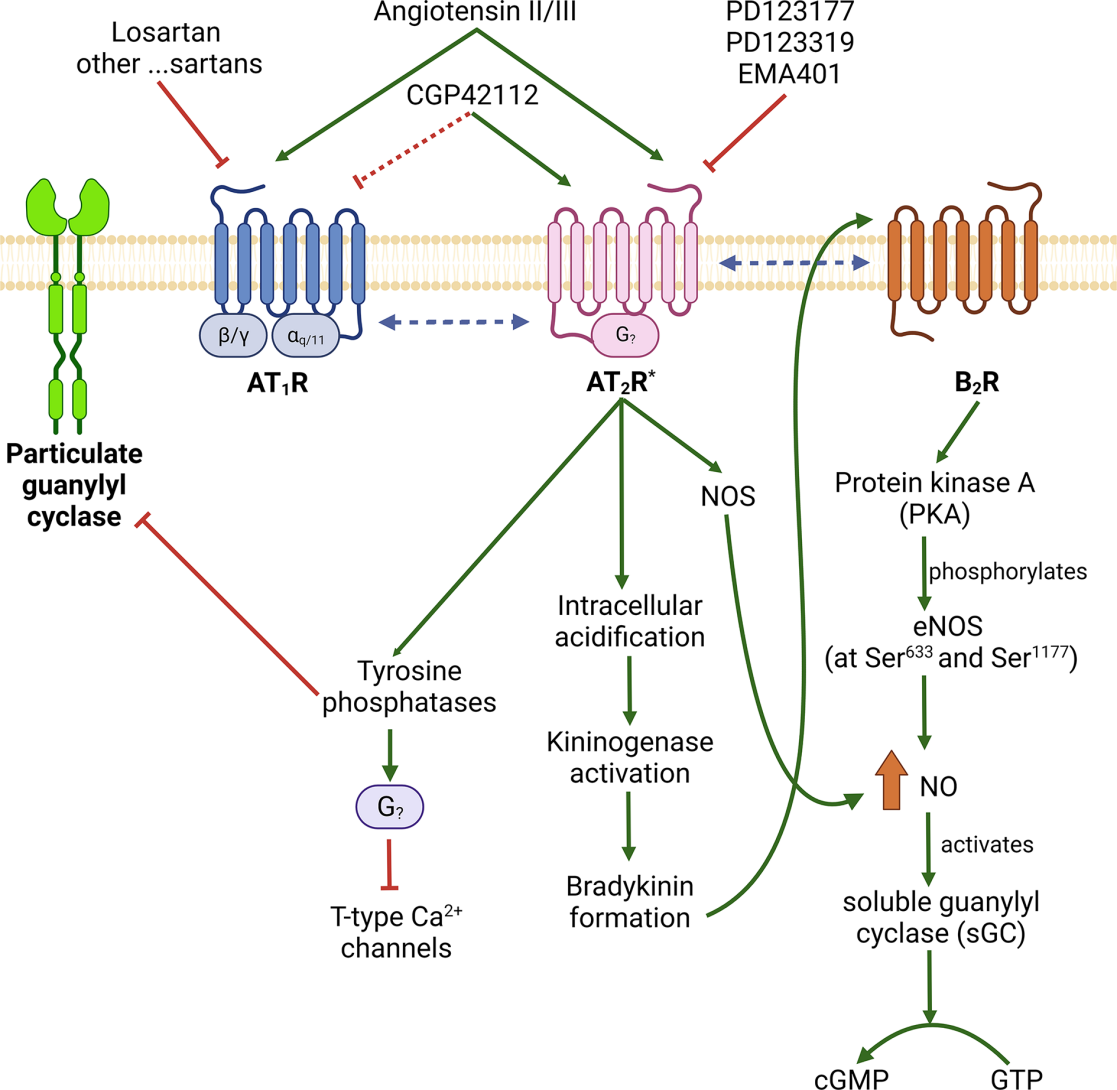
Activation and inhibition of AT_1_ and AT_2_ Ang II receptors The pharmacology and signaling pathways of the AT_1_R are well established [51] and not described in this figure, which instead focuses on several of the proposed pharmacology and signaling pathways of the AT_2_R. Agonist ligands for the AT_2_R shown in the figure are Ang II and III, as well as CGP42112. Not shown are three other AT_2_R selective agonists; compound 21 (C21) [52], ß-Pro^7^ Ang III [15], and pNH_2_Phe^6^Ang II [32,35,53]. The putative pathways shown are from Brechler et al. inhibition of particulate guanylyl cyclase via tyrosine phosphatase in a G protein-independent manner [23], increased production of NO and cGMP by direct activation of NOS and soluble guanylate cyclase [54], via stimulation of bradykinin formation [26,55], which stimulates B_2_ bradkyinin receptor mediated eNOS activation, formation of NO which stimulates synthesis of cyclic guanosine monophosphate (cGMP) by soluble guanylate cyclase [27], or with subsequent activation of the B_2_ receptor; and via dimerization of AT_2_R and bradykinin B_2_ receptor which also increases NO synthesis and cGMP formation [29], and heterodimerization with the AT_1_R to inhibit its function [56]. *Signaling pathways have been reported to be both G protein-dependent and G protein-independent.

What is known so far is that there are a variety of signaling pathways for the AT_2_R. Some of the AT_2_R signaling pathways are reported to involve the G_i/o_ protein [18–20], or an unspecified G protein that is not G_i/o_ [21], while others appear to be G protein independent [22–25]. Main pathways include stimulation of several phosphatases; stimulation of bradykinin formation [26], which activates B_2_ bradykinin receptors [27], which activates nitric oxide synthase (NOS), leading to cGMP formation; inhibition of particulate guanylate cyclase activity via phosphotyrosine phosphatase (PTP) activation [23], heterodimerization with AT_1_ receptors to inhibit their function [28], as well as heterodimerization with the bradykinin B_2_ receptor [29] (Figure 1). Additionally, the AT_2_R has been reported to both activate [30–32] and inhibit [33,34] nuclear factor kappa B (NF-κB) signaling. Ruiz-Ortega et al. [35] also stated that CGP42112 acted upon the AT_2_R to stimulate NF-κB signaling; however, they used 10 µM CGP42112 so it is not possible to reach any conclusions regarding its actions as an AT_2_R agonist or antagonist from this study. Noting the ambiguities regarding the agonistic and antagonistic properties for which CGP42112 has been attributed, Rompe et al. [33] stated ‘Both features rendered CGP42112A a problematic tool for research and prevented its development for clinical use’.

Finally, AT_2_R activation can result in tissue remodeling leading to apoptosis, inhibition of hyperplasia in pathological situations, and reduction of cardiac inflammation and fibrosis [11,36,37].

## Mischaracterization of CGP42112

As seen in the literature, CGP42112 is referred to by three different names: CGP42112, CGP42112A, or GP42112B. The ‘A’ and ‘B’ versions of this compound indicate different salt forms. CGP42112A refers to the hydrochloride salt, while CGP42112B represents the trifluoroacetate salt [38]. However, there is likely minimal differential pharmacological effects between these two salt forms of CGP42112.

The uncertainty that surrounds the classification of CGP42112 increased when publications started referring to it as a partial agonist of the AT_2_R. Although it is hard to pinpoint the exact time when this mischaracterization began, our research dates it back to 1993, when Timmermans et al. wrote a review and stated that CGP42112A possessed partial agonistic effects due to it being a modified peptide but did not provide any data or citations proving such [10]. Macari et al. also published the concept as ‘a possibility’, but, again, without supporting data [39]. Based upon their expertise and strong reputation in the RAAS field, their postulation was taken as fact, whereupon later publications started to incorrectly refer to the compound as a partial agonist of the AT_2_R (Supplementary Table S2). However, based on empirical studies that looked at the agonistic properties of CGP42112, noted above, there is evidence showing that it is in fact a full agonist of the AT_2_R [5,6,15], which is consistent with other papers that interpret it as a full agonist based on the near equivalency of the responses to CGP42112 and Ang II at concentrations that give a near maximal response [4,40].

Yet another factor that may contribute to the ambiguity of agonism of CGP42112 at the AT_2_R is cell type-specific responses. Thus, the different responses mediated by AT_2_R on NG108-15, N1E-115, PC12W, NIH 3T3 fibroblasts, neonatal rat neurons, rabbit proximal tubule epithelial cells, and AT_2_R transfected cells is another confounder in characterizing the agonistic effects of CGP42112. The extreme example of antithetical AT_2_R signaling is the report that AT_2_R in NIH3T3 cells [24] and PC12W cells (specifically CGP42112) [41] can both stimulate and inhibit ERK1/2 depending on whether the cells are quiescent or stimulated by nerve growth factor (NGF), respectively.

Another source of the misconception that CGP42112 is a partial agonist is the misinterpretation of the concentration response curve (CRC) of CGP42112 relative to that of Ang II by Hansen et al. [19]. Despite showing an arbitrary maximum response of 100% of ^35^S-GTPγS incorporation to both Ang II and CGP42112 at concentrations up to 1 µM, a reduction in the response at 10 µM CGP42112 probably led Hansen et al., to mischaracterize CGP42112 as a partial agonist.

Further adding to the misconception of the efficacy of CGP42112 is that while a potent agonist at the AT_2_R, it does not interact significantly with the AT_1_R until its concentration approaches the micromolar range. Thus, part of the reason for the mischaracterization seems to involve the role of CGP42112 at different concentrations. It has been reported that at low concentration (typically nM) [42], the compound functions as an agonist, but as the concentration increases (typically µM and above), it gains antagonistic properties at the AT1R [38], which is where some of the confusion lies. As concentration increases, the empirical studies mentioned above show that CGP42112 remains a full agonist at the AT_2_R, while it also begins to bind to the AT_1_R, acting in an antagonistic manner [3,38,43].

A comparison between two papers by Macari et al. reveals a major inconsistency in their classification of CGP42112 [38,39]. As noted above, Macari et al. characterized CGP42112 as a weak AT_1_R antagonist [38]. However, upon blockade of the RAAS with an angiotensin-converting enzyme (ACE) inhibitor, CGP42112 was reported to gain partial agonistic properties at the AT_1_R [39] completely contradicting the conclusion of their 1993 paper, with respect to the efficacy of CGP42112 at the AT_1_R. With the supposition in mind, that CGP42112 was a partial agonist at the AT_1_R, Macari et al. suggested that the same might be true at the AT_2_R [39]. Based upon the way in which the experiments were conducted, it is believed that the interpretation of the 1994 experiment was flawed, since it utilized losartan to saturate the AT_1_R, precluding any effects of CGP42112B on the AT_1_R, as it would have been unable to bind to it. This contradicts the findings from their 1993 paper, where they stated that CGP42112B acts as an AT_1_R antagonist at higher concentrations when the RAAS is not inhibited. Since the conditions at which each experiment was conducted are different, an exact classification of the compound based upon the comparison of the two studies cannot be achieved. Further complicating matters, there is a single report of CGP42112 acting as an antagonist of Ang II at the AT_2_R [44]. The concentrations of CGP42112 used in that study were 0.1 to 1 µM, opening up the possibility that the antagonistic effects were occurring at the AT_1_R. Additionally, they state in their manuscript, ‘In addition, 10^−6^ M of the peptide AT_2_ antagonist CGP-42112A with some agonistic properties was also used’, without providing any citation. Of note, these authors also used the classical AT_2_R antagonist, PD123319, to block activation of their AT_2_R-mediated response.

## CGP42112 is a full agonist at the AT_2_R

As of 2021 (based on our ‘CGP42112’ PubMed search), CGP42112 has most recently been consistently referred to as an AT_2_R agonist. However, in most cases, a full dose-response curve comparison with Ang II was not done (Supplementary Table S3), so no conclusion as to whether CGP42112 is a full agonist can be made from such studies. Nonetheless, the definitive studies of Brechler et al. [5] and Widdop et al. [6] showing equivalent AT_2_-agonist efficacy CRCs of Ang II and CGP42112 along with the near identical CRCs of Ang III and CGP42112 reported by Del Borgo et al. [15]; the anomalous concentration response data for CGP42112 showing equiefficacy as an AT_2_ agonist with Ang III [19]; the near identical maximal agonist responses of Ang II and CGP42112 at the AT_2_R [4,40]; the absence of any empirical data showing partial agonism of CGP42112 at the AT_2_R, together provide compelling evidence that CGP42112 is a full agonist.

## Perspective

This analysis strives to remove the stigma of CGP42112's mischaracterization as a partial agonist. This ambiguity has caused CGP42112 to fall out of favor as a tool to study the importance of the AT_2_R in the RAAS. Compound 21 (C21), which has yet to be shown to have a clinical application, has been used in nearly 200 studies (40 of which were reported between 2020 and 2022) using the search terms ‘C21 and AT_2_’, or ‘compound 21 and AT_2_’, or ‘C21 and angiotensin’, or ‘compound 21 and angiotensin’, in PubMed, while CGP42112 has only been reported to have been used once each in 2020 and 2021, with no publications so far in 2022 using the search terms ‘CGP42112 and AT_2_’, or ‘CGP42112 and angiotensin’, in PubMed.

Prior to its virtual abandonment as a tool to study AT_2_R function, CGP42112 was used to show that AT_2_R stimulation can further lower blood pressure when used in combination with an AT_1_R antagonist [13,14] and that the AT_2_R promotes natriuresis [45]. Given that Lee et al. showed that CGP42112 had neuroprotective effects mediated by the AT_2_R while C21 was ineffective as a neuroprotectant [46], necessitates correct characterization of CGP42112 as a full AT_2_R agonist so that it can continue to be used to assess the receptor’s functionality in concert with other AT_2_R agonists and antagonists [47–49].

An additional concern for the use of CGP42112 as a tool to study the RAAS has been that it is a peptide, in contrast with C21, which is a small molecule that can be administered orally. However, development of novel formulations of peptide drugs using nanotechnology has enabled their administration in oral forms with increased *in vivo* stability and bioavailability [50]. Thus, CGP42112, by virtue of its greater potency as an AT_2_R agonist, can serve as a more powerful AT_2_R agonist as well as an alternative to C21. To avoid the risk of confounding by an idiosyncratic action of a single AT_2_R agonist, future studies should use multiple AT_2_R agonists to better understand the function of the AT_2_R

It is critical to revisit previous reports that mischaracterized CGP42112 to eliminate some of the ambiguities that have plagued our ability to fully appreciate AT_2_R’s function in the RAAS.

## Supplementary Material

Supplementary Tables S1-S3

## Data Availability

N/A

## Abbreviations

ACE: angiotensin-converting enzyme
Ang: angiotensin
AT_1_R: angiotensin II type 1 receptor
AT_2_R: angiotensin II type 2 receptor
cGMP: cyclic guanosine monophosphate
CRC: concentration response curve
eNOS: endothelial nitric oxide synthase
GPCR: G-protein-coupled receptor
NF-κB: nuclear factor κB
NGF: nerve growth factor
NOS: nitric oxide synthase
PTP: phosphotyrosine phosphatase
RAAS: renin–angiotensin–aldosterone system
