# Supplementary material for: CGP42112: the full AT2 receptor agonist and its role in the renin–angiotensin–aldosterone system: no longer misunderstood
Source: Clin Sci (Lond). 2022 Nov 3;136(21):1513–33. doi: 10.1042/CS20220261 (PMC9638965; doi:10.1042/CS20220261)
Supplement: Supplementary Tables S1-S3 [file CS-2022-0261_supp.pdf]

Authors	Year	Nomenclature	PubMed ID (PMID)	Characterization	Source	Source PubMed ID (PMID)
Whitebread, S. et al.	1989	CGP42112A	2775266	AT2 selective ligand	radioligand binding data (in vitro); did not address efficacy	
Criscione, L. et al.	1990	CGP42112A	1705629	Angiotensin IIB [AT1R] antagonist	experimental data (in vitro)	
Bumpus, F. M. et al.	1991	CGP42112A	2022414	AT2 antagonist (with caveat pending functional characterization)	uncited	
Tsutsumi, K. and Saavedra, J. M.	1991	CGP42112A	1986946	AT2 antagonist	miscited Whitebread, 1989; did not address efficacy	2775266
Ji, H. et al.	1991	CGP42112A	1996080	AT2 antagonist	miscited Whitebread, 1989; characterization of CGP42112 as an Ang II receptor antagonist was based upon inhibition of AT1R-mediated effects	2775266
Jaiswal, N. et al.	1991	CGP42112A	2035693	AT2 antagonist	miscited Whitebread, 1989	2775266
Tsutsumi, K. et al.	1991	CGP42112A	1717294	AT2 antagonist	miscited Whitebread, 1989	2775266
Felix, D. et al.	1991	CGP42112A	2045157	AT2 antagonist	miscited Whitebread, 1989	
Tallant, E. A. et al.	1991	CGP42112A	2045160	AT2 antagonist	uncited	
Sumners, C. et al.	1991	CGP42112A	1881896	AT2 antagonist	uncited	
Tsutsumi, K. and Saavedra, J. M.	1991	CGP42112A	1887916	AT2 antagonist	miscited Whitebread, 1989; did not address efficacy	2775266
Millan, M. A. et al.	1991	CGP4211A	1763058	AT2 antagonist	uncited	
Zhou, J. et al.	1992	CGP42112A	1279763	AT2 antagonist	uncited	
Rowland, N. E. et al.	1992	CGP42112A	1393612	AT2 antagonist	uncited	
Beresford, M. J. and Fitzsimons, J. T.	1992	CGP42112B	1418958	AT2 antagonist	cited Whitebread, 1989 but only saying "AT2R bind to CGP42112B"	2775266
Ambühl, P. et al.	1992	CGP42112A	1333623	AT2 antagonist	uncited	
Ouali, R. et al.	1992	CGP42112A	1327066	AT2 antagonist	uncited	
Viard, I. et al.	1992	CGP42112A	1426267	AT2 antagonist	uncited	
Janiak, P. et al.	1992	CGP42112A	1452289	AT2 antagonist	uncited; mischaracterized the AT2R antiproliferative response as being AT2R antagonism	
Jaiswal, N. et al.	1992	CGP42112A	1735595	AT2 antagonist	uncited	
Zarahn, E.D. et al.	1992	CGP42112A	1313856	AT2 antagonist	uncited	
Millet, D. et al.	1992	CGP42112A	1375911	AT2 antagonist	uncited	
Timmermans, P. B. et al.	1992	CGP42112A	1290617	peptide antagonist	miscited Whitebread, 1989	2775266
Barnes, K. L. et al.	1993	CGP42112A	8095842	AT2 antagonist	uncited	
Aiyar, N. et al.	1993	CGP42112A	8434027	AT2 antagonist	uncited	
Le Noble, F. A. et al.	1993	CGP42112A	8447504	AT2 antagonist	uncited; misinterpreted the antiproliferative effects of AT2R agonism to be AT1R antagonism effect of CGP42112 differed from PD123319	
Kumagai, H. et al.	1993	CGP42112A	8384603	AT2 antagonist	uncited	
Naville, D. et al.	1993	CGP42112A	8477848	AT2 antagonist	uncited	
Jaiswal, N. et al.	1993	CGP42112A	8496814	AT2 antagonist	uncited	
Zhou, J. et al.	1993	CGP42112A	8389324	AT2 antagonist	uncited	
Montiel, M. et al.	1993	CGP42112A	8240673	AT2 antagonist	uncited	
Edwards, R. M. and Stack, E. J.	1993	CGP42112A	8394907	AT2 antagonist	uncited	
Bensoussan, M. et al.	1993	CGP42112A	8282006	AT2 antagonist	uncited	
Cooney, A. S. and Fitzsimons, J. T.	1993	CGP42112B	8311944	AT2 antagonist	uncited	
Pörsti, I. et al.	1993	CGP42112	7510856	AT2 antagonist	uncited	
Chatziantoniou, C. and Arendshorst, W. J.	1993	CGP42112	8285218	AT2 antagonist	uncited	
Madhun, Z. T. et al.	1993	CGP42112A	8469770	AT2 antagonist	uncited	
Timmermans, P. B. et al.	1993	CGP42112A	8372104	AT2 antagonist	uncited or cited papers in which agonist properties of CGP42112 were misinterpreted as antagonists based upon misunderstanding of AT2 receptor function	
Santos, R. A. and Campagnole-Santos, M. J.	1994	CGP42112A	8087084	AT2 antagonist	uncited	

Goldfarb, D. A. et al.	1994	CGP42112A	8254815	AT2 antagonist	uncited	
Corriu, C. et al.	1994	CGP42112A	8141364	AT2 antagonist	uncited	
Lokuta, A. J. et al.	1994	CGP42112A	8106454	AT2 antagonist	uncited	
Hogarty, D. C. et al.	1994	CGP42112A	8180789	AT2 antagonist	uncited; likely high dose antagonism of AT1R response; effect differed from PD123319	
Santos, R. A. et al.	1994	CGP42112A	7850477	AT2 antagonist	uncited	
Fow, J. E. et al.	1994	CGP42112A	7914065	AT2 antagonist	uncited	
Fontes, M. A. et al.	1994	CGP42112A	7882013	AT2 antagonist	uncited	
Chatziantoniou, C. et al.	1994	CGP42112	7700009	AT2 antagonist	uncited	
Rowland, N. E. et al.	1994	CGP42112A	8092325	AT2 weak antagonist	uncited	
Freeman, E.J. and Tallant, E. A.	1994	CGP42112	7998990	AT2 antagonist	miscited Whitebread, 1989	2775266
Chappell, M.C. et al.	1995	CGP42112A	7479311	AT2 antagonist	uncited	
de Gasparo, M. et al.	1995	CGP42112	7737728	AT2 antagonist	cited Bumpus, 1995	2022414
Song, K. et al.	1995	CGP42112B	9072342	AT2 antagonist	uncited	
el Ghissassi, M. et al.	1995	CGP42112A	7611515	AT2 antagonist	uncited	
Pelet, C. et al.	1995	CGP42112A	7556378	AT2 antagonist	uncited	
Sum, C. S. and Cheung W. T.	1995	CGP42112	8584570	AT2 antagonist	uncited	
Peral de Bruno, M. and Coviello, A.	1995	CGP42112A	8617893	AT2 antagonist	uncited	
Braszko, J.	1996	CGP42112A	8787210	AT2 antagonist	uncited	
Schäfer, F. et al.	1996	CGP42112A	8680854	AT2 antagonist	uncited	
Santos, R. A. et al.	1996	CGP42112A	8613263	AT2 antagonist	uncited	
Oliveira, D. R. et al.	1996	CGP42112A	8641737	AT2 antagonist	cited Timmermans, 1993	8372104
Saridogan, E. et al.	1996	CGP42112B	9239679	AT2 antagonist	uncited	
Cox, B. E. et al.	1996	CGP42112A	8760177	AT2 antagonist	uncited	
Sum, C. S. et al.	1996	CGP42112	8832081	AT2 antagonist	cited Timmermans, 1993	8372104
Saridogan, E. et al.	1996	CGP42112B	8675601	AT2 antagonist	uncited	
Phillips, M. I. et al.	1996	CGP42112A	8899885	AT2 antagonist	uncited	
Lee, W. J. et al.	1996	CGP42112A	8899891	AT2 antagonist	uncited	
Tierney, M. et al.	1997	CGP42112	9000463	AT2 antagonist	miscited Nahmias and Strosberg, 1995	7667895
Inwang, E. R. et al.	1997	CGP42112A	9155046	AT2 antagonist	uncited	
Braszko, J. J. et al.	1997	CGP42112A	9407709	AT2 antagonist	uncited	
Ruan, X. et al.	1997	CGP42112	9062366	AT2 antagonist	uncited	
Muller, C. et al.	1997	CGP42112A	9421301	AT2 antagonist	cited de Gasparo, 1995; CGP42112 response was consistent with an agonist effect but PD123319 response was anomalous	7737728
Dubey, R. K. et al.	1998	CGP42112	9761285	AT2 antagonist	uncited	
Braszko, J. J. et al.	1998	CGP42112A	9990655	AT2 antagonist	miscited Whitebread, 1989; likely high dose antagonism of AT1R response	2775266
Gohlke, P. et al.	1998	CGP42112A	9453327	AT2 antagonist	uncited	
Martineau, D. et al.	1999	CGP42112	10535686	AT2 antagonist	experimental data (in vivo)	
Quan, A. and Baum, M.	1999	CGP42112A	10342788	AT2 antagonist	uncited	
Watanabe, T. et al.	2000	CGP42112A	10661500	AT2 antagonist	uncited	
Ruiz-Ortega, M. et al.	2000	CGP42112	10864918	AT2 antagonist	uncited; experimental data at 10 µM which would be interacting with AT1R	
Cerra, M. C. et al.	2001	CGP42112	11178878	AT2 antagonist	uncited	
Fabiani, M. E. et al.	2001	CGP42112	11572794	AT2 antagonist	uncited	
Chassagne, C. et al.	2002	CGP42112A	11880254	AT2 antagonist	cited Ruiz-Ortega, 2000; Sumners, 1991	10864918, 1881896
Breigeiron, M. K. et al.	2002	CGP42112	11971660	AT2 antagonist	uncited	
Muscella, A. et al.	2002	CGP42112	12010639	AT2 antagonist	uncited	
Inada, Y. et al.	2002	CGP42112	12570021	AT2 antagonist	uncited	
Imbrogno, S. et al.	2003	CGP42112	12819273	AT2 antagonist	cited de Gasparo, 1995; Cerra, 2001	7737728, 11178878
Bagi, E. E. et al.	2003	CGP42112	12834880	AT2 antagonist	miscited Whitebread, 1989; Timmermans, 1993	2775266, 8372104
de Arruda Camargo, L. A. et al.	2003	CGP42112A	13679246	AT2 antagonist	uncited	
de Arruda Camargo, L. A. et al.	2003	CGP42112A	14642649	AT2 antagonist	uncited	

Mima, E. G. O. et al.	2004	CGP42112A	15003832	AT2 antagonist	uncited	
Abrão Saad, W. et al.	2004	CGP42112A	15109940	AT2 antagonist	uncited	
Pelegrini-da-Silva, A. et al.	2005	CGP42112A	15802196	AT2 antagonist	cited Timmermans, 1993	8372104
Akhavan, M. M. et al.	2005	CGP42112A	16165374	AT2 antagonist	uncited	
Saad, W. A. et al.	2005	CGP42112A	16198010	AT2 antagonist	uncited	
Doller, A. et al.	2009	CGP42112	19246637	AT2 antagonist	uncited	

Authors	Year	Nomenclature	PubMed ID (PMID)	Characterization	Source	Source PubMed ID (PMID)
Timmermans, P. B. et al.	1992	CGP42112A	1290617	partial agonist	uncited	
Timmermans, P. B. et al.	1993	CGP42112A	8372104	partial agonist	inference based upon CGP42112 being a modified peptide	
Macari, D. et al.	1994	CGP42112B	7957590	partial agonist	inference based upon CGP42112 actions on AT1R	
Stoll, M. et al.	1995	CGP42112	7860748	partial agonist	experimental data (in vitro) and cited Brechler, 1993; Brechler, 1994a; Brechler, 1994b; Criscione, 1990; Le Noble, 1993	8385791, 7525002, 7953293, 1705629, 8447504
Groblewski, T. et al.	1997	CGP42112A	8999867	partial agonist	experimental data (in vitro) using mutated AT1R	
Wolf, G. et al.	1997	CGP42112A	9276721	partial agonist	uncited	
Hansen, J. L. et al.	2000	CGP42112	11055978	partial agonist	based upon inability to act as an agonist at the AT1R despite data indicating that CGP42112 was a full agonist at the AT2R*	
Bautista, R. et al.	2001	CGP42112A	11566953	partial agonist	uncited	
Warnecke, C. et al.	2001	CGP42112	11692164	partial antagonist**	uncited	
Berry, C. et al.	2001	CGP42112A	11709400	partial agonist	cited Hansen, 2000	11055978
Schuijt, M. P., Saxena, P. R., and Jan Danser, A. H.	2002	CGP42112	12019289	partial agonist	cited Carey, 2001	11751702
Bagi, E. E., Fekete, E., and Lénárd, L.	2003	CGP42112	12834880	partial agonist	cited Martens, 1996	8756008
Montiel-Herrera, M., Miledi, R., and García-Colunga, J.	2006	CGP42112A	16288466	partial agonist	cited Ferguson, 2001	11446443
Lemarié, C. A. and Schiffrin, E. L.	2010	CGP42112A	19861349	partial agonist	uncited	
Verdonk, K. et al.	2012	CGP42112A	22348403	partial agonist	cited Stoll, 1995	7860748
Verdonk, K. et al.	2012	CGP42112A	22802221	partial agonist	uncited	

Authors	Year	Nomenclature	PubMed ID (PMID)	Characterization*	Source	Source PubMed ID (PMID)
Buisson, B. et al.	1992	CGP42112	1324194	agonist	experimental data (in vitro)	
Brechler, V. et al.	1993	CGP42112	8385791	full agonist	experimental data (in vitro)	
Kambayashi, Y. et al.	1993	CGP42112A	8227011	agonist	experimental data (in vitro)	
Brechler, V. et al.	1994	CGP42112	7525002	agonist	uncited	
Brechler, V. et al.	1994	CGP42112	7953293	agonist	uncited	
Näveri, L. et al.	1994	CGP42112	8263056	agonist	uncited	
Kalenga, M. K. et al.	1994	CGP42112A	8141364	agonist	uncited	
Israel, A. et al.	1995	CGP42112A	8665267	agonist	experimental data (in vitro)	
Nahmias, C. et al.	1995	CGP42112	7532401	agonist	uncited	
Chabre, O. et al.	1995	CGP42112A	7867605	agonist	uncited	
Mancina, R. et al.	1996	CGP42112A	8626829	agonist	uncited	
Martens, J. R. et al.	1996	CGP42112	8756008	agonist	experimental data (in vitro)	
Ozawa, Y. et al.	1996	CGP42112	8920914	full agonist	experimental data (in vitro)	
Tsuzuki, S. et al.	1996	CGP42112A	8941361	agonist	uncited	
Laredo, J. et al.	1997	CGP42112	9039134	agonist	uncited	
Liakos, P. et al.	1997	CGP42112	9357777	agonist	uncited	
Belloni, A. S. et al.	1998	CGP42112	9553751	agonist	uncited	
Jin, X. H. et al.	1998	CGP42112A	9688688	agonist	uncited	
Tanabe, A. et al.	1998	CGP42112	9761377	agonist	uncited	
Naruse, M. et al.	1998	CGP42112	9806212	agonist	uncited	
Zwart, A. S.	1998	CGP42112	9884070	agonist	uncited	
Li, J. Y. et al.	1998	CGP42112	9888501	agonist	uncited	
Tanabe, A. et al.	1999	CGP42112	10361432	agonist	uncited	
Côté, F. et al.	1999	CGP42112	10531378	agonist	uncited	
Barber. M. N.	1999	CGP42112	10567191	agonist	experimental data (in vivo)	
Chamoux, E. et al.	1999	CGP42112	10599741	agonist	uncited	
Chassagne, C. et al.	2000	CGP42112A	10696069	agonist	uncited	
Takekoshi, K. et al.	2000	CGP42112	10833449	full agonist	cited Israel, 1995; Buisson, 1995	8665267
Mifune, M. et al.	2000	CGP42112A	11082154	agonist	uncited	
Ruiz-Ortega, M. et al.	2000	CGP42112	10864918	agonist	cited Timmermans, 1993	8372104
Johansson, B. et al.	2001	CGP42112A	11352819	agonist	experimental data (in vivo)	
Takekoshi, K. et al.	2001	CGP42112	11416030	agonist	cited Israel 1995; Buisson, 1992; Buisson, 1995; Brechler, 1993	8665267, 1324194, 7829501, 8385791
Hines, J. et al.	2001	CGP42112A	11454929	agonist	cited Brechler, 1993	8385791
Fischer, J. W. et al.	2001	CGP42112A	11530112	agonist	uncited	
Carey, R. M. et al.	2001	CGP42112A	11751702	agonist	experimental data (in vivo)	
Widdop, R. E. et al.	2002	CGP42112	12364356	full agonist	experimental data (in vitro)	
Baranov, D. and Armstead, W. M.	2002	CGP42112A	12490010	agonist	uncited	
Rhinehart, K. et al.	2003	CGP42112A	12424093	agonist	uncited	
Ewert, S. et al.	2003	CGP42112A	12689346	agonist	experimental data	
Duke, L. M. et al.	2003	CGP42112	12847115	agonist	cited de Gasparo, 2000; Barber, 1999	10977869, 10567191
Benndorf, R. et al.	2003	CGP42112A	12881481	agonist	uncited	
Ichihara, A. et al.	2003	CGP42112A	14714584	agonist	uncited	
Gendron, L. et al.	2003	CGP42112	12464615	agonist	experimental data (in vitro)	
Wu, L. et al.	2004	CGP42112A	14684844	agonist	uncited	
Liu, R. et al.	2004	CGP42112A	14744924	agonist	uncited	
Li, X. C. and Widdop, R. E.	2004	CGP42112	15197103	agonist	experimental data (in vivo)	
Hakam, A. C. and Hussain, T.	2005	CGP42112A	15596573	agonist	cited Carey, 2001; Barber, 1999	11751702, 10567191
Kim, M. P. et al.	2005	CGP42112A	15817699	agonist	cited Côté, 1999	10531378
Lemos, V. S. et al.	2005	CGP42112A	16116331	agonist	uncited	
Hakam, A. C. and Hussain, T.	2006	CGP42112	16380464	agonist	uncited	

Hakam, A. C. and Hussain, T.	2006	CGP42112	16618840	agonist	uncited	
Beaudry, H. et al.	2006	CGP42112	16740968	agonist	uncited	
Ereso, A. Q. et al.	2007	CGP42112	17903725	agonist	uncited	
van de Wal, R. M. A. et al.	2007	CGP42112A	18049304	agonist	cited Brechler, 1993; Hines, 2001	8385791, 11454929
Vaajanen, A. et al.	2008	CGP42112A	18223252	agonist	uncited	
Kilian, P. et al.	2008	CGP42112	18326001	agonist	uncited	
Tani, T. et a.	2008	CGP42112A	18543083	agonist	uncited	
Cervenka, L. et al.	2008	CGP42112A	18551014	agonist	uncited	
Sasaoka, T. et al.	2008	CGP42112A	18670361	agonist	uncited	
Gao, L. et al.	2008	CGP42112	18768398	agonist	uncited	
Kanome, T. et al.	2008	CGP42112A	18971559	agonist	uncited	
McCarthy, C. A. et al.	2009	CGP42112	19246705	agonist	uncited	
Bosnyak, S. et al.	2010	CGP42112	20128808	agonist	cited Barber et al., 1999; Li and Widdop, 2004	10567191, 15197103
Song, R. et al.	2010	CGP42112	20032120	agonist	cited Brechler, 1993	8385791
Sabuhi, R. et al.	2010	CGP42112A	20668101	agonist	uncited	
Wu, X. et al.	2010	CGP42112A	20718738	agonist	uncited	
Sabuhi, R. et al.	2011	CGP42112A	21209001	agonist	uncited	
Raffai, G. et al.	2011	CGP42112	21803946	agonist	uncited	
Girasole, A. E. et al.	2011	CGP42112A	21865545	agonist	uncited	
Abadir, P. M. et al.	2011	CGP42112	21288138	agonist	used CGP42112 and PD123319 to show a reduction in phosphorylated STAT3 by the AT2R	
Lee, S. et al.	2012	CGP42112	22920387	agonist	experimental data (in vivo/ in vitro)	
Yang, J. et al.	2012	CGP42112	22504846	agonist	uncited	
McCarthy, C. A. et al.	2012	CGP42112	23090772	agonist	uncited	
McCarthy, C. A. et al.	2013	CGP42112	23224511	agonist	cited Widdop, 2002	12364356
Abdulla, M. A. and Johns, E. J.	2013	CGP42112	23751965	agonist	uncited	
Gao, S. et al.	2013	CGP42112A	23791669	agoinst	uncited	
Ali, Q. et al.	2013	CGP42112A	23823602	agonist	experimental data (in vivo)	
Wang, D.	2013	CGP42112	24112447	agonist	experimental data (in vivo/ in vitro)	
Kljajic, S. T. et al.	2013	CGP42112	24161533	agonist	uncited	
Park, M. H. et al.	2013	CGP42112	24289788	agonist	uncited	
Xu, Y. et al.	2013	CGP42112A	24340072	agonist	uncited	
Abdulla, M. A. and Johns, E. J.	2014	CGP42112	24279649	agonist	uncited	
Veron, J. B. et al.	2014	CGP42112A	24388688	agonist	cited Buisson,1992	1324194
Wainford, R.D.	2014	CGP42112	24447624	agonist	cited Gao, 2008; Abdulla, 2014	18768398, 24279649
Longman, M.R.	2014	CGP42112	24475092	agonist	uncited	
Umschweif, G. et al.	2014	CGP42112A	24957202	agonist	uncited	
Park, Y. A. et al.	2014	CGP42112A	25014541	agonist	uncited	
Wei, Y. et al.	2014	CGP42112	25100281	agonist	uncited	
Yoshida, T. et al.	2014	CGP42112	25112871	agonist	uncited	
Chow, B. S. et al.	2014	CGP42112	24429402	agonist	uncited	
Lu, J. et al.	2015	CGP42112	25446015	agonist	uncited	
Miura, S. I. et al.	2015	CGP42112	25496380	agonist	uncited	
Gong, W. K. et al.	2015	CGP42112	25562714	agonist	uncited	
Luo, H. et al.	2015	CGP42112A	25687731	agonist	uncited	
Itinteang, T. et al.	2015	CGP42112	25713419	agonist	uncited	
Del Borgo, M. et al.	2015	CGP42112	26186568	agonist	experimental data (in vitro)	
Zhu, L. et al.	2015	CGP42112	26163449	agonist	uncited	
Ma, C. Y. and Yin, L.	2016	CGP42112	27630693	agonist	uncited	
Raffai, G. and Lombard J. H.	2016	CGP42112	27676088	agonist	uncited	
Schwengel, K. et al.	2016	CGP42112	26983606	agonist	cited McCarthy, 2009 and 2012	19246705, 23090772
Littlejohn, N. K. et al.	2016	CGP42112A	27477281	agonist	cited Hines, 2001	11454929
Abdulla, M. A. and Johns, E. J.	2017	CGP42112	27820727	agonist	uncited	

Yang, Y. et al.	2018	CGP42112	29289466	agonist	uncited	
Zhu, Y. et al.	2019	CGP42112A	31857626	agonist	uncited	
Wang, Y. et al.	2020	CGP42112	32259090	agonist	uncited	
Bhat, S.A., et al.	2021	CGP42112A	34529240	agonist	uncited	

Supplemental Table 1. Papers suggesting that CGP42112 is an AT_2R antagonist

Starting from its initial characterization in 1989 by Whitebread et al. up until 2009, a total of 94 papers refer to CGP42112 as an AT_2R antagonist.

Supplemental Table 2. Papers suggesting that CGP42112 is an AT_2R partial agonist

Starting in 1992 (Timmermans et al.), up until 2012, a total of 16 papers refer to CGP42112 as an AT_2R partial agonist.

* A comparison between Ang III and CGP42112 for formation of 35S-GTP reported by Hansen et al., 2000 indicated a high potency for Ang III (EC50 = 880 pM with an R_{max} value approximating 100%, based upon a recalculation of the data presented in their Figure 5C). The concentration response curve for CGP42112 however, was anomalous, so it was not possible to derive a reliable EC50 or R_{max} value (not shown). Constraining the R_{max} value to 100% for CGP42112 over a concentration range of 10 pM to 1 µM, yielded an EC50 value of 83 nM when R_{max} limited to 100% suggesting that CGP42112 was less potent than Ang III, but possibly more efficacious.

** In 2001, Warnecke et al. referred to CGP42112 as a partial antagonist, which suggests that it is also a partial agonist.

Supplemental Table 3. Papers suggesting that CGP42112 is an AT_2R agonist or full agonist

Starting in 1992 (Buisson et al.), up until 2021, a total of 106 papers refer to CGP42112 as either an AT_2R agonist (102) or full agonist (4).

*For the purpose of this paper, the agonist characterization was assumed to infer a full agonist, however in most cases, no data distinguishing full versus partial agonism was provided.
